# Resveratrol Chemosensitizes TNF-β-Induced Survival of 5-FU-Treated Colorectal Cancer Cells

**DOI:** 10.3390/nu10070888

**Published:** 2018-07-12

**Authors:** Constanze Buhrmann, Mina Yazdi, Bastian Popper, Parviz Shayan, Ajay Goel, Bharat B. Aggarwal, Mehdi Shakibaei

**Affiliations:** 1 Musculoskeletal Research Group and Tumour Biology, Chair of Vegetative Anatomy, Institute of Anatomy, Faculty of Medicine, Ludwig-Maximilian-University Munich, Pettenkoferstrasse 11, D-80336 Munich, Germany; constanze.buhrmann@med.uni-muenchen.de (C.B.); mina.yazdi@alumni.ut.ac.ir (M.Y.); 2 Biomedical Center, Core facility animal models, Ludwig-Maximilian-University Munich, D-82152 Martinsried, Germany; bastian.popper@med.uni-muenchen.de; 3 Department of Parasitology, Faculty of Veterinary Medicine, University of Tehran, Tehran 141556453, Iran; pshayan@ut.ac.ir; 4 Center for Gastrointestinal Research; Center for Translational Genomics and Oncology, Baylor Scott & White Research Institute and Charles A Sammons Cancer Center, Baylor University Medical Center, Dallas, TX 75246, USA; Ajay.Goel@BSWHealth.org; 5 Anti-inflammation Research Institute, San Diego, CA 92126, USA; bbaggarwal@gmail.com

**Keywords:** resveratrol, colorectal cancer, cancer stem cells, TNF-β, 5-fluorouracil, alginate culture

## Abstract

**Objective:** Resveratrol, a safe and multitargeted natural agent, has been linked with inhibition of survival and invasion of tumor cells. Tumor Necrosis Factor-β (TNF-β) (Lymphotoxin α) is known as an inflammatory cytokine, however, the underlying mechanisms for its pro-carcinogenic effects and whether resveratrol can suppress these effects in the tumor microenvironment are poorly understood. **Methods:** We investigated whether resveratrol modulates the effects of 5-Fluorouracil (5-FU) and TNF-β on the malignant potential of human colorectal cancer (CRC) cells (HCT116) and their corresponding isogenic 5-FU-chemoresistant derived clones (HCT116R) in 3D-alginate tumor microenvironment. **Results:** CRC cells cultured in alginate were able to migrate from alginate and the numbers of migrated cells were significantly increased in the presence of TNF-β, similar to TNF-α, and dramatically decreased by resveratrol. We found that TNF-β promoted chemoresistance in CRC cells to 5-FU compared to control cultures and resveratrol chemosensitizes TNF-β-induced increased capacity for survival and invasion of HCT116 and HCT116R cells to 5-FU. Furthermore, TNF-β induced a more pronounced cancer stem cell-like (CSC) phenotype (CD133, CD44, ALDH1) and resveratrol suppressed formation of CSC cells in two different CRC cells and this was accompanied with a significant increase in apoptosis (caspase-3). It is noteworthy that resveratrol strongly suppressed TNF-β-induced activation of tumor-promoting factors (NF-κB, MMP-9, CXCR4) and epithelial-to-mesenchymal-transition-factors (increased vimentin and slug, decreased E-cadherin) in CRC cells. **Conclusion:** Our results clearly demonstrate for the first time that resveratrol modulates the TNF-β signaling pathway, induces apoptosis, suppresses NF-κB activation, epithelial-to-mesenchymal-transition (EMT), CSCs formation and chemosensitizes CRC cells to 5-FU in a tumor microenvironment.

## 1. Introduction

The worldwide incidence of colorectal cancer (CRC) has risen to approximately 1.2 million new cases and 0.6 million deaths annually [1]. Although in high-income countries, the disease incidence has stabilized due to enhanced screening [1], treatment of CRC still poses a clinical challenge as 5-year survival rates are around 65% depending on tumor location, stage of tumor detection and treatment [2].

A key feature to support and modulate colon cancer progression is the result of complex interaction of tumor cells with their microenvironment [3,4,5]. It is recognized that chronic inflammation alters the tumor microenvironment thus supporting development and progression of cancer [6,7]. Activation of the pro-inflammatory NF-κB-signaling pathway represents a central event in the tumor-development progress and enhances tumor progression [8]. Several pro-inflammatory mediators that have been shown to alter the tumor microenvironment, including members of the Tumor Necrosis Factor (TNF)-superfamily, are regulated by the transcription factor NF-κB [9,10]. TNF-α itself is produced in the tumor microenvironment and regulates the communication between tumor cells, their surrounding stromal cells and the extracellular matrix in several cancers, acting as an autocrine and paracrine growth factor stimulating further expression of other growth factors [11,12].

Whereas TNF-α acts as an initiator and modulator of tumorigenesis has been intensively studied [13], the role of another member of the TNF-superfamily during cancer development, Lymphotoxin-α (alias TNF-β) needs to be further characterized [13,14]. It is known that TNF-β binds to membrane-bound lymphotoxin-β heteromers and signals through lymphotoxin-β receptor (LTBR) [15], and may activate both the canonical and non-canonical NF-κB pathway [16,17,18]. Furthermore, it has been shown that TNF-β induces apoptosis and inflammatory signals similar to TNF-α [19]. Studies on ovarian cancer cells demonstrated that TNF-β overexpression is commonly found in different ovarian cancer subtypes, and that the LTBR is expressed ubiquitously in ovarian cancer cells as well as cancer-associated fibroblasts [14]. Additionally, in ovarian cancer TNF-β has been shown to promote tumor-stromal cells interaction in the tumor microenvironment [14].

It is believed that tumor progression, resistance and metastasis are driven by a subpopulation of cells with stem cell like characteristics [20,21]. These cancer stem cells (CSC) are defined by their ability of self-renewal, formation of differentiated cancer cells, and initiation of cancer progression [21,22]. First described for hematologic malignancies, over the past years there has been an accumulating evidence of CSC also in solid cancers including CRC [22,23,24]. Previous studies have proposed that CSC are responsible for the maintenance of the tumor microenvironment and its progression is markedly influenced by cross talk of CSC with the tumor microenvironment, leading to enhanced secretion of chemokines and cytokines [25,26]. It is now widely believed that targeting the cancer stem cell microenvironment may be the key in the development of novel therapeutic strategies [3].

Besides surgery, adequate chemotherapy with 5-Fluorouracil alone or in combination with other chemotherapeutics (such as oxaliplatin) is the gold standard of CRC therapy [27,28]. Limited success of treatment response and high recurrence are hypothesized to be due to insensitivity of CSC to treatment, promoting survival of tumors and re-initiating tumor growth and metastasis [29,30]. Recently, several studies have shown that addition of natural substances as adjuvants to conventional chemotherapy may specifically target CSCs and subsequently sensitize chemoresistant CRC cells to chemotherapeutics, by modulating a wide variety of targets such as pro-inflammatory pathways and tumor transcription factors [31,32,33,34,35].

The natural polyphenol resveratrol (3,5,41-trihydroxystilbene) is a phytoalexin produced in several plants such as grapes, peanuts and various herbs, protecting them against UV-radiation, oxidative stress and fungal infections [36,37,38]. Previous studies have shown that resveratrol acts as a multi-targeted component and possesses anti-inflammatory and anti-tumor activities in CRC [39,40,41,42]. Furthermore, resveratrol has been shown to exert a chemosensitization effect on cancer cells when applied in combination with standard chemotherapeutics, such as 5-FU, by up-regulation of intercellular junctions and focal adhesion molecules, blocking epithelial-to-mesenchymal-transition (EMT), suppressing inflammatory pathways and increasing apoptosis [41,43,44].

Thus, in the present study we investigated the modulatory effect of resveratrol in a TNF-β-mediated inflammatory tumor microenvironment on malignity of 5-FU resistant and non-resistant CRC cells during early stages of tumorigenesis in monolayers and 3D-alginate culture model. 

## 2. Materials and Methods

### 2.1. Antibodies

Antibodies to ALDH1, CD44, CD133 were purchased from antibodies online and antibody to β-Actin was from Sigma-Aldrich Chemie (Munich, Germany). Antibodies to MMP-9 and activated caspase-3 were purchased from R&D Systems (Heidelberg, Germany). Antibodies to CXCR4 were purchased from Abcam PLC (Cambridge, UK) and to phospho-specific p65 (NF-κB) were from Cell Technology (Beverly, MA, USA). Anti-vimentin, anti-slug and anti-E-cadherin were purchased from Santa Cruz Biotechnology (Santa Cruz, CA, USA). Secondary alkaline phosphatase–linked sheep anti-mouse and sheep anti-rabbit antibodies for immunoblotting were from EMD Millipore (Schwalbach, Germany) and secondary antibodies for fluorescence labeling from Dianova (Hamburg, Germany). The antibodies were applied at dilutions suggested by the manufacturers.

### 2.2. Growth Media, Cytokines and Chemicals

Cell culture growth medium was prepared as previously described [41,44]. All experiments were performed in serum-starved growth medium supplement only with 3% FCS. Epon was obtained from Plano (Marburg, Germany). Alginate, 5-Fluorouracil and resveratrol were purchased from Sigma. Resveratrol was prepared in ethanol as 100 mM stock solution and further diluted in cell culture medium to prepare working solutions. The final maximum content of ethanol in cultures was less than 0.1% and this concentration was also used as a basal control. 5-FU was prepared in Dimethylsufoxide (DMSO) as 1000 µM stock solution and further diluted to reach final concentration of less than 1% in cell cultures. TNF-β and polyclonal rabbit anti-TNF-β were obtained as described [45] and additionally TNF-β and TNF-α, both purified to homogeneity with specific activity of 50 million units/mg, were a kind gift from Genetech, Inc. (South San Francisco, CA, USA).

### 2.3. Cell Lines and Cell Culture

The human colon cancer cell line HCT116 was purchased from the European Collection of Cell Cultures (Salisbury, UK). From this cell line, a 5-Fluorouracil (5-FU) resistant cell line (HCT116R) was generated as previously described [32]. Cell culture was performed in tissue culture flasks in growth medium and in a humidified incubator at 37 °C in an atmosphere of 95% air and 5% CO_2_ and the medium was changed every two to three days. Cells were passaged at 80–90% confluency using trypsin/EDTA.

### 2.4. Alginate Tumor Microenvironment Culture

Alginate tumour microenvironment culture for 3-dimensional cultivation of CRC cells has been previously described [33,41,44]. Briefly, the colorectal cancer cells were re-suspended in alginate (2% in 0.15 M NaCl) at a concentration of 1 × 10^6^/mL, added drop wise into a 100 mM CaCl_2_ solution at ambient temperature (were the alginate polymerized into stable beads) and washed with 0.15 M NaCl solution and with serum-starved medium before starting treatment. All subsequent treatments were carried out in serum starved medium.

### 2.5. Invasion Assay

Starved HCT116 and HCT116R cells were cultured in 3-dimensional alginate culture as described above to investigate invasion and migration capacity. In an additional set of experiments, CRC cells were left untreated, treated with 10 ng/mL TNF-α, 10 ng/mL TNF-β, treated with 5 µM resveratrol alone, 5-FU (0.1 or 1 nM) alone, or a combination of 1 nM 5-FU and either 10 ng/mL TNF-α or 10 ng/mL TNF-β, or a combination of 5 µM resveratrol and 1 nM 5-FU alone or in combination with either 10 ng/mL TNF-α or 10 ng/mL TNF-β. At day 10, cells migrated through the alginate matrix and adhered at the bottom of the petri dish were fixated with Karnowsky and stained with toluidine blue for 10 min. The number of adhered colonies was quantified by counting all colonies under a light microscope (Zeiss, Jena, Germany).

### 2.6. Immunofluorescence

Immunofluorescent investigation was performed on HCT116 and HCT116R monolayer cultures that migrated from alginate cultures after 10 days with anti-ALDH1 and anti-CD44. Alginate cultures were left untreated or treated with either 5 µM resveratrol alone, 10 ng/mL TNF-α, 10 ng/mL TNF-β or a combination of 5 µM resveratrol and either 10 ng/mL TNF-α or 10 ng/mL TNF-β. Immunofluorescence labelling was performed as previously described [41]. Briefly, adhered cells that migrated from the alginate matrix were fixated with methanol, rinsed with PBS and incubated with PBS/1% bovine serum albumin (BSA) for 10 min. Subsequently, cells were incubated overnight in a humid chamber at 4 °C with primary antibodies diluted in PBS/1% BSA. After washing three times with PBS, secondary antibodies were incubated for 1.5 h, nuclear staining performed with DAPI and cells covered with fluoromount mountant and examined under a fluorescent microscope (Leica, Darmstadt, Germany). Quantification of positively stained ALDH1 or CD44 cells and of apoptotic cells (DAPI) was performed by scoring 300 cells from 10 different microscopic fields. The values were compared to the control, and statistically-significant values were labelled with *p* < 0.05 are designated by an asterisk (*); *p* < 0.01 by two asterisks (**).

### 2.7. Quantification of Apoptosis with DAPI

DAPI (4, 6-Diamidino-2-phenylindole, Sigma) nuclear staining assay was performed to assess the number of apoptotic changes induced by TNF-α, TNF-β, 5-Fluorouracil (5-FU) and resveratrol and their combination in HCT116 and HCT116R cells as previously described [41]. Briefly, cell were seeded on glass plates, and either left untreated, treated with either 5 µM resveratrol alone, 10 ng/mL TNF-α, 10 ng/mL TNF-β, 0.1 and 1 nM 5-FU or a combination of 0.1 and 1 nM 5-FU with either 10 ng/mL TNF-α or 10 ng/mL TNF-β, or a combination of 5 µM resveratrol and 1 nM 5-FU alone or with either 10 ng/mL TNF-α or 10 ng/mL TNF-β for 48 h and fixed with methanol. DAPI solution was applied for 10 min. in the dark and cells were evaluated under a fluorescence microscope (Leica, Germany) and visualized. Quantification of apoptotic cells was performed by scoring 800 cells from 20 different microscopic fields. All values were compared to the control, and statistically-significant differences were labelled with *p* < 0.05 (*); *p* < 0.01 (**).

### 2.8. Ultrastructural Investigations

In an additional set of experiments, alginate beads from HCT116 and HCT116R CRC cells were either left untreated, treated with 5 µM resveratrol alone, 10 ng/mL TNF-α, 10 ng/mL TNF-β, 1 nM 5-FU or a combination of 1 nM 5-FU with either 10 ng/mL TNF-α or 10 ng/mL TNF-β, or a combination of 5 µM resveratrol and 1 nM 5-FU alone or additionally with either 10 ng/mL TNF-α or 10 ng/mL TNF-β for 10 days. Subsequently, cells were fixed with Karnowsky fixative and the ultrastructure of cells were evaluated as described previously [33,44]. Statistical evaluation of apoptotic cells was performed by counting 300 cells from 20 different microscopic fields. All values were compared to the control, and statistically-significant differences were labelled with *p* < 0.05 (*); *p* < 0.01 (**).

### 2.9. Western Blot Analysis

HCT116 and HCT116R CRC cells were cultured in alginate bead culture and either left untreated, treated with either 5 µM resveratrol alone, 10 ng/mL TNF-α, 10 ng/mL TNF-β, 0.1 and 1 nM 5-FU or a combination of 0.1 and 1 nM 5-FU with either 10 ng/mL TNF-α or 10 ng/mL TNF-β, or a combination of 5 µM resveratrol and 1 nM 5-FU alone or with either 10 ng/mL TNF-α or 10 ng/mL TNF-β for 10 days and immunoblotting performed as previously described [46].

### 2.10. Statistical Analysis

Experiments were performed three times as individual experiments with three individual replicates. For statistical analysis, a Wilcoxon–Mann–Whitney test was applied. Data were shown as mean values ± SD or SEM and were compared by one-way, or two-way or a three-way ANOVA using SPSS Statistics, if the normality test passed (Kolmogorov–Smirnov test). A *p* value of <0.05 was considered to establish statistically significant differences.

## 3. Results

The aim of this study was to examine the potential role of TNF-β to induce an inflammatory microenvironment to promote CRC cell malignancy alone or during treatment with 5-FU in human CRC cells (HCT116 and HCT116R) in a 3D-alginate tumor microenvironment. We used a well-characterized 3D-alginate tumour microenvironment culture model that allows examination of the early, initial steps of tumorigenesis such as invasion and migration of cancer cells. Furthermore, we investigated the modulatory effects of resveratrol on TNF-β-mediated inflammatory signaling in the treatment of CRC either alone or in combination with 5-FU.

### 3.1. Resveratrol Chemosensitizes CRC Cells to 5-FU and Suppresses Invasion in TNF-β-, Similar to TNF-α-Induced Pro-Inflammatory Alginate Tumor Microenvironment Cultures

**Figure 1:**
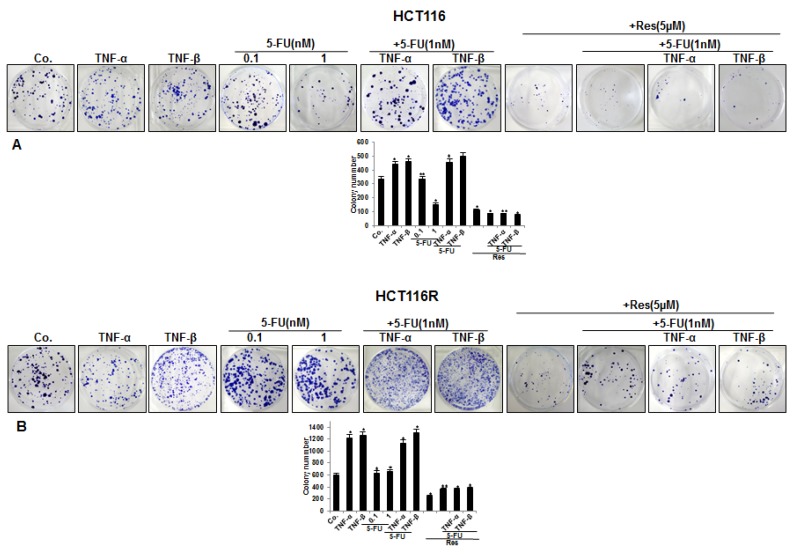
Effect of resveratrol and/or 5-FU on CRC cell migration induced by TNF-β or TNF-α in inflammatory microenvironment. Serum-starved HCT116 (**A**) and HCT116R (**B**) were cultured in alginate culture and treated as described in detail in “Section 2”. Invasive colonies were stained with toluidine blue and the number of emigrated spheroids was quantified after 10 days in culture. Each experiment was repeated at least three times and experimental values were compared with the control and statistically significant values with *p* < 0.05 were designated by an asterisk (*) and *p* < 0.01 were designated by two asterisks (**).

To evaluate the effect of resveratrol and/or 5-FU on TNF-β-induced invasion capacity of CRC cells in a 3D inflammatory tumor microenvironment, HCT116 and HCT116R cells (1 × 10^6^/mL) were cultured in an alginate-based matrix, treated as described in detail in Material and Methods and the capacity of migration and invasion was determined through evaluation of colony-formation with toluidine blue staining after 10 days. Treatment of the CRC cells with 5-FU (0.1, 1 nM) alone significantly blocked migration of HCT116 cells through the alginate in a dose dependent manner (Figure 1A). Interestingly, it should also be stated here that there was no effect of 5-FU on the 5-FU resistant cells (HCT116R), even after treatment with 1 nM dose (Figure 1B), highlighting that HCT116R cells were indeed resistant to 5-FU. Moreover, we found that the combined treatment of 5-FU with TNF-β similar to TNF-α synergistically enhanced the invasion ability of HCT116 and HCT116R cells in comparison to the individual compound (Figure 1A,B). However, HCT116R migrated significantly more under the same condition compared to HCT116 cells from the alginate beads, indicating that TNF-β promotes an inflammatory microenvironment under chemotherapeutic treatment and increases the malignant potential of human CRC cells for the 5-FU resistant cells (Figure 1B). Next, we investigated whether resveratrol modulates the enhanced migration of the CRC cells (HCT116 and HCT116R) by combined treatment of 5-FU and/or TNF-β, similar to TNF-α, through 3D alginate-based culture microenvironment. As shown in Figure 1A,B, we found that treatment of CRC cells (HCT116 and HCT116R) with resveratrol (5 µM) alone blocked (*p* < 0.05) or resveratrol (5 µM) and co-treatment with 5-FU (1 nM) and TNF-β (10 ng/mL) or TNF-α (10 ng/mL) enhanced dramatically inhibition of the invasion ability of HCT116 and HCT116R cells through the alginate-based matrix compared to untreated cells (Figure 1A,B). Quantification of cell colonies confirmed these results. Taken together, these results indicate that TNF-β as a pro-inflammatory cytokine can activate tumor cells in the microenvironment medium that in turn induces tumor cell activation, promoting progression and expanded metastatic patterns, increasing thereby the malignancy of the cancer cells. Inhibition of this inflammatory pathway by resveratrol induces signaling and functional changes towards sensitizing CRC cells to 5-FU treatment.

### 3.2. Resveratrol Suppresses TNF-β-, Similar to TNF-α-Induced Formation of CSCs in Migrated CRC Cells Monolayer Culture as Revealed by Immunofluorescence Microscopy

**Figure 2:**
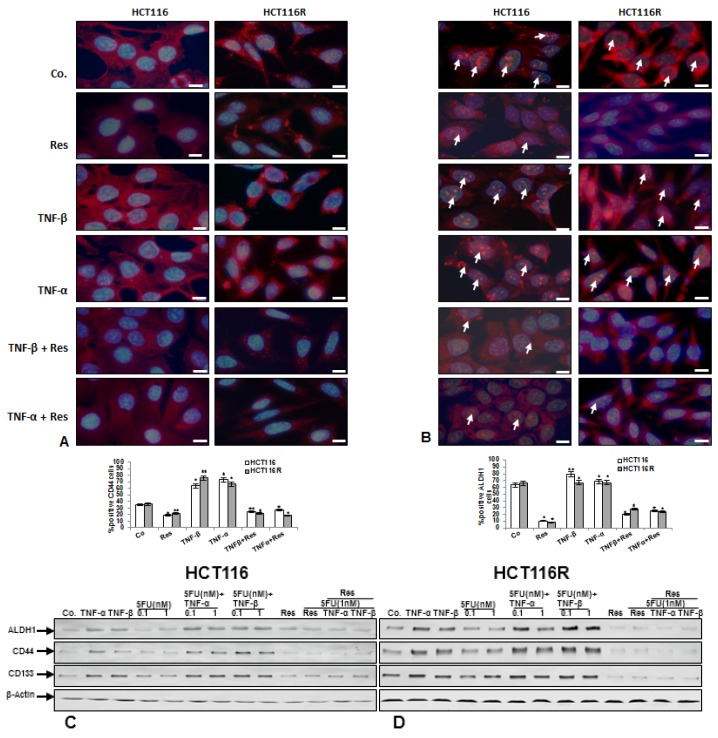
(**A**,**B**): Effect of resveratrol on CSC formation in TNF-β-induced inflammatory microenvironment. Serum-starved HCT116 and HCT116R were cultured in alginate culture and treated as described in detail in “Section 2”. Invasive cells from the alginate that adhered at the bottom of the petri dish and formed colonies were subjected to immunolabeling with primary antibodies against CD44 (**A**) and ALDH1 (**B**) followed by incubation with rhodamine-coupled secondary antibodies and counterstaining with DAPI to visualize cell nuclei. Images shown are representative of three different experiments. Magnification 600×; bar = 30 nm. The number of positively stained cells was quantified by counting 300 cells from ten different microscopic fields of view. The values were compared to the control and statistically-significant values with *p* < 0.05 and significant values are marked with an asterisk (*) and *p* < 0.01 were designated by two asterisks (**). (**C**,**D**): Effect of resveratrol and/or 5-FU on cancer stem cell formation induced by TNF-β in CRC cells in inflammatory microenvironment culture. Serum-starved HCT116 (**C**) and HCT116R (**D**) in alginate culture were treated as described in detail in “Section 2”. After 10 days whole cell lysates were prepared and western blotting performed with antibodies against ALDH1, CD44 and CD133. Western blots shown are representative of three independent experiments. The housekeeping protein β-actin served as a positive loading control in all experiments.

Because a TNF-β-induced inflammatory microenvironment promotes migration of CRC cells, we investigated whether TNF-β increased formation of cancer stem cells (CSCs) and whether resveratrol could modulate TNF-induced CSC formation. Furthermore, a large body of literature indicates that formation and proliferation of CSC-like cells are induced by inflammatory cells, cytokines and growth factors in the tumor microenvironment [47,48,49,50,51,52]. To detect CSC formation and behavior under the abovementioned conditions, we used migrated cells from alginate beads. For this reason, the HCT116 and HCT116R (1 × 10^6^/mL) were cultured in an alginate-based tumor microenvironment, treated as described in detail in Material and Methods and cells that had migrated through the alginate beads and formed adhered monolayer culture on the bottom of the glass dish were examined. These monolayer cultures were subjected to immunofluorescence labeling with primary antibodies for colon CSC markers (CD44 and ALDH1). We found, that moderate expression of CD44 and ALDH1 was detected in basal control in HCT116 and HCT116R cells (Figure 2A,B). Interestingly, in contrast, TNF-β, similar to TNF-α increased the number of CD44 and ALDH1 positive cells in HCT116 and HCT116R cells compared to that in control monolayer cultures (Figure 2A,B), indicating the important role of TNF-β-mediated inflammatory medium to induce malignant potential of human CRC cells by promotion of CSC formation. Furthermore, in the presence of resveratrol and/or TNF-β and/or TNF-α, cells showed marked down-regulation of CD44 and ALDH1 positive cells (Figure 2A,B), demonstrating the prominent targeting effect of resveratrol on CSCs, as an important anti-carcinogenic mechanism of resveratrol. Quantification of CD44- and ALDH1-positively labeled HCT116 and HCT116R cells confirmed the immunofluorescence results (Figure 2A,B).

Resveratrol blocks TNF-β-induced CSC-formation and chemosensitizes CSC to 5-FU in pro-inflammatory alginate tumor microenvironment cultures.

To confirm the immune-cytochemical observations that TNF-β-induced CSCs formation in an alginate tumor microenvironment and that resveratrol can modulate this effect and additionally to demonstrate the chemosensitization effect of resveratrol to 5-FU on CSC markers (CD133, CD44 and ALDH1) expression, western blotting analysis was performed (Figure 2C,D). Alginate cultures of HCT116 and their corresponding 5-FU-resistant clones were either left untreated, or were treated as described in detail in Material and Methods. As shown in Figure 2C,D, control alginate cultures of HCT116 and HCT116R colon cancer cells showed basal expression of CSC markers (ALDH1, CD44 and CD133). We found that TNF-β, like TNF-α treatment individually, or in combination (TNF-β or TNF-α with 5-FU), significantly increased CSCs as shown by up-regulation of specific CSCs markers (CD44, CD133 and ALDH1). We found further that 5-FU treatment individually suppressed CSCs in HCT116 cell population as revealed by decreased expression of specific CSCs markers (Figure 2C). However, there was minimal or no effect of 5-FU on HCT116R cells, even after treatment with 1 nM (Figure 2D), underlining that HCT116R cells are resistant to 5-FU. In contrast, immunoblotting analysis results showed clearly that resveratrol alone or in combination with 5-FU or with TNF-β or with TNF-α induced marked down regulation of CD133, CD44 and ALDH1 in HCT116 and HCT116R cells in comparison to control in 3D alginate cultures (Figure 2C,D).

### 3.3. Resveratrol Potentiates 5-FU-Mediated Apoptosis in TNF-β-Induced Survival of CRC Cells in Monolayer Cultures

**Figure 3:**
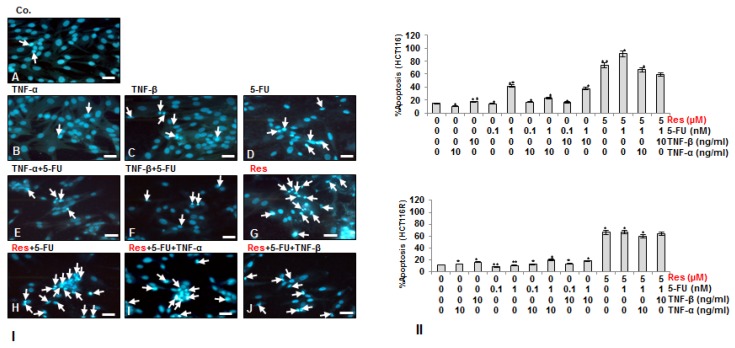
Effect of resveratrol and/or 5-FU on apoptosis induced by TNF-β in HCT116 and HCT116R cells. Serum-starved HCT116 (**I**,**II**) and HCT116R (**II**) were cultured on glass plates and treated as described in detail in “Section 2” for 72 h and DAPI nuclear staining assay was performed to determine the amount of apoptotic nuclei. **II:** The number of the apoptotic nuclei was quantified by counting 800 cells from 20 microscopic fields. The examination was performed in triplicate and the results are provided as mean values with standard deviations *p* < 0.05 are designated by an asterisk (*); *p* < 0.01 by two asterisks (**). Magnification: 400×.

To examine whether the suppressing effect of resveratrol and/or 5-FU on TNF-β-induced cell viability is related to the promoting of apoptosis, HCT116 (Figure 3I,II) and HCT116R (Figure 3II) cells were either left untreated or treated as described in detail in Material and Methods and stained with DAPI to reveal apoptotic bodies (Figure 3). We found that treatment with TNF-β, similar to TNF-α alone, or 5-FU (0.1 nM) did not induce significant chromatin condensation, resulting in 19%, 10% and 14.5% of apoptotic nuclei in HCT116 and of 13%, 11% and 7% in HCT116R similar to untreated control cultures (15% and 10% respectively) (Figure 3I,II). Contrary to this, in HCT116 cells, the number of apoptotic nuclei markedly increased to 41% by treatment with 1 nM 5-FU, whereas in HCT116R cells treatment with 1 nM 5-FU resulted only in 9% apoptotic cells, similar to untreated control. Further, in HCT116 cells combinational treatment of either TNF-β or TNF-α with 5-FU (0.1, 1 nM) lead to an apoptotic increase of 20% and 39% and of 19% and 27% respectively. In contrast to HCT116, in HCT116R cells, combinational treatment of either TNF-β or TNF-α with 5-FU (0.1, 1 nM) only marginally increased the number of apoptotic nuclei by 10% and 9% and by 2% and 6% respectively. Resveratrol treatment alone significantly increased the number of apoptotic nuclei in HCT116 (73%) and HCT116R cells (65%). Furthermore, co-treatment of resveratrol with 5-FU (1 nM) alone or as combinational treatment with 5-FU (1 nM) and TNF-β or TNF-α significantly increased nuclear fragmentation and apoptosis in all tumor cells (Figure 3I,II). Hereby, in HCT116 apoptosis increased by 90%, 60%, and 69%, and in HCT116R by 68%, 61%, and 58% respectively (Figure 3I,II). Indeed, HCT116R cells increased in the surviving cell population upon treatment with 5-FU and TNF-β similar to TNF-α, but not with resveratrol or the combined treatment, indicating that resveratrol may sensitize chemoresistant cells to 5-FU (Figure 3I,II). These results confirmed the results in Figure 1, highlighting that resveratrol not only sensitizes HCT116 and HCT116R colon cancer cells to 5-FU-induced apoptosis, but is further able to do this in a pro-inflammatory environment, blocking TNF-β-induced survival of CRC cells.

### 3.4. Resveratrol Suppresses TNF-β- Similar to TNF-α-Enhanced Survival in with 5-FU-Treated CRC Cells by Apoptosis in Alginate Tumor Microenvironment

**Figure 4:**
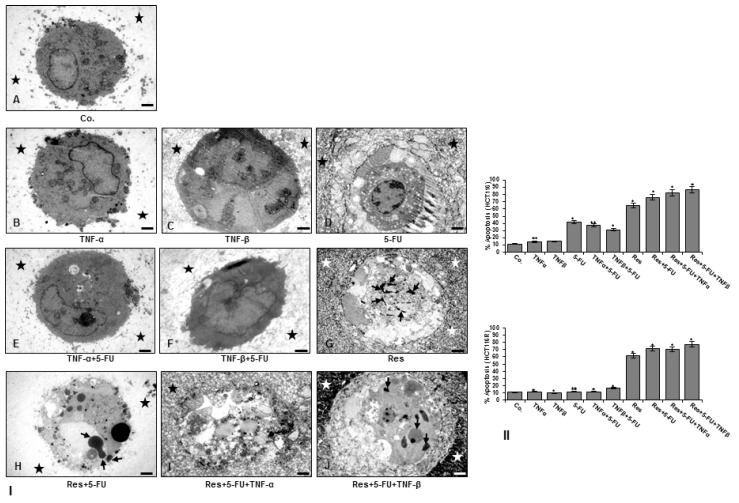
Ultrastructural demonstration of cell viability and apoptosis of CRC cells after treatment with resveratrol and/or 5-FU in TNF-β-induced inflammatory microenvironment. Serum-starved HCT116 (**I**,**II**) and HCT116R (**II**) were cultured in alginate culture and treated as described in detail in “Section 2” for 10 days and spheroid formation and apoptotic induction investigated. Magnification: ×5000, bar = 1 μM. **II:** The number of apoptotic cells was quantified by counting 300 cells from 20 different microscopic fields. Values were compared to the control, and statistically-significant values were labelled with *p* < 0.05 are designated by an asterisk (*); *p* < 0.01 by two asterisks (**).

Because resveratrol can reduce (modulate) the colony formation and invasion effects of TNF-β and enhance 5-FU-effects in 3D alginate beads, HCT116 (Figure 4I,II) and HCT116R (Figure 4II) cells were either left untreated, treated as described in detail in Material and Methods and apoptotic cells were investigated by transmission electron microscopy evaluation of colonogenic formation in alginate beads after 10 days. Ultrastructural analysis showed that TNF-β, similar to TNF-α alone or with 5-FU did not induce significant chromatin condensation (Figure 4I(B,C,E,F)) similar to control of HCT116 (Figure 4I(A)); however, treatment with 5-FU or resveratrol alone or combined treatment with resveratrol, 5-FU and TNF-β or TNF-α in HCT116 and HCT116R (not shown) markedly induced degeneration of cell organelles and appearance of multiple vacuoles, chromatin condensation with prominent signs of apoptosis (Figure 4I(D,G–J)). Interestingly, it was noted that there was little or no effect of 5-FU with or without TNF-β on HCT116R cells, indicating that HCT116R cells are resistant to 5-FU and underlining the important role of TNF-β-induction on the malignant potential of human CRC cells and the chemosensitisation potential of resveratrol even in a TNF-β-induced pro-inflammatory microenvironment (Figure 4I,II). Statistical evaluation of the ultrastructural samples highlighted the main effects of combined TNF-β, 5-FU and resveratrol treatment in promoting and synergistically enhancing apoptosis in both HCT116 and HCT116R cells compared to control tumor cultures (Figure 4I,II) suggesting that resveratrol may inhibit inflammation and sensitize 5-FU in the chemoresistant cell line even under pro-inflammatory condition with TNF-β. Thus, these results suggest that TNF-β causes an inflammatory and pro-carcinogenic malignant microenvironment for HCT116 and 5-FU chemoresistant cells, where the cells are less sensitive to chemotherapeutic agents such as 5-FU. However, resveratrol alone or in combination with 5-FU may represent a potential treatment option for 5-FU resistant colon cancer cells even under pro-inflammatory condition.

### 3.5. Resveratrol Blocks TNF-β-Induced NF-κB Activation and NF-κB-Dependent Gene Products Involved in Migration, Metastasis and Apoptosis of CRC Cells and Chemosensitizes to 5-FU in Pro-Inflammatory Tumor Microenvironment Cultures

**Figure 5:**
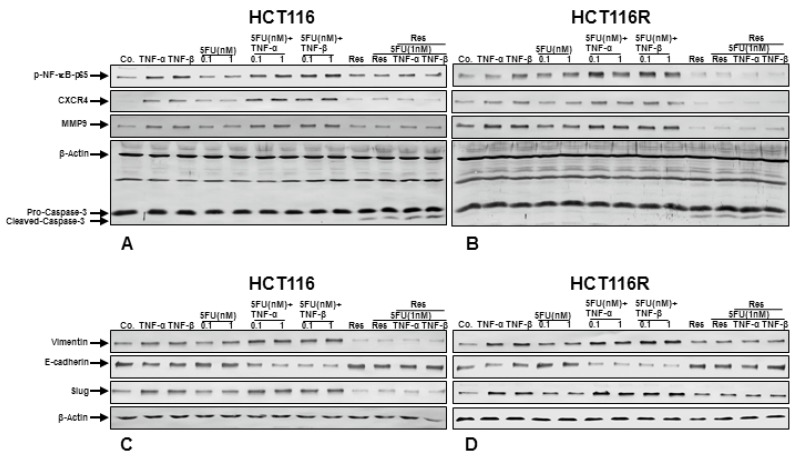
(**A**,**B**): Effect of Resveratrol and/or 5-FU on NF-κB activation and NF-κB-regulated gene end-products involved in apoptosis, metastasis induced by TNF-β in HCT116 and HCT116R in inflammatory microenvironment. Alginate cultures of HCT116 (**A**) and HCT116R (**B**) were treated for 10 days as described in detail in “Section 2”. Immunoblotting with whole cell lysates was performed with antibodies against p65-NF-κB, CXCR4, MMP-9 and cleaved-caspase-3. Western blots shown are representative of three independent experiments. The housekeeping protein β-actin served as a positive loading control in all experiments. (**C**,**D**): Effect of resveratrol and/or 5-FU on TNF-β-induced epithelial-to-mesenchymal transition of CRC cells in tumor microenvironment cultures. Colorectal cancer cells in alginate culture were treated as described in Materials and Methods. After 10 days whole cell lysates of HCT116 (**C**) and HCT116R (**D**) were subjected to western blotting with antibodies against vimentin, E-cadherin and slug. Western blots shown are representative of three independent experiments. The housekeeping protein β-actin served as a positive loading control in all experiments.

To explore the underlying molecular mechanism of how resveratrol suppresses TNF-β-induced malignancy of CRC cells and chemosensitizes to 5-FU, we investigated whether the effects of resveratrol on CRC cells in TNF-β-induced pro-inflammatory alginate tumor microenvironments was associated with the inhibition of NF-κB activation and NF-κB-regulated gene products involved in tumor metastasis. Indeed, it has been reported that cytokines, like TNF-α, execute their pro-inflammatory effects primarily through activation of NF-κB, regulating the expression of inflammatory genes involved in invasion and metastasis [52,53]. Serum-starved CRC cells (HCT116 and HCT116R) in alginate beads were either left untreated or treated as described in Material and Methods, and as shown in Figure 5, we examined the expression of the NF-κB and NF-κB-regulated gene products that are involved in invasion (MMP-9) and metastasis (CXCR4). The results of western blot analysis showed a basal expression of above mentioned protein expression in control alginate cultures of CRC cells and this expression was significantly increased in the presence of TNF-β, similar to TNF-α treatment individually, or in combination (TNF-β or TNF-α with 5-FU) (Figure 5A,B). We further found that 5-FU alone suppressed the expression of NF-κB, MMP-9 and CXCR4 in HCT116 cell population (Figure 5A), but there was a minimal or no effect of 5-FU on HCT116R cells, even after treatment with 1 nM (Figure 5B), highlighting that HCT116R cells are chemoresistant to 5-FU. In opposite, western blotting analysis results showed clearly that resveratrol alone or in combination with 5-FU or with TNF-β or with TNF-α substantially down-regulated the mentioned proteins expression in both HCT116 and HCT116R cells in alginate tumor microenvironment cultures (Figure 5A,B). All together, these findings further strengthen the essential role of resveratrol in modulating TNF-β and/or 5-FU-induced NF-κB-regulated tumor metastasis promoting gene products in CRC cells.

We further investigated whether resveratrol can modulate NF-κB-dependent gene products involved in apoptosis (cleavage of caspase-3) of CRC cells in alginate tumor microenvironment. As shown in Figure 5A,B, resveratrol induced caspase-3 cleavage in HCT116 (Figure 5A) and HCT116R (Figure 5B). The combined treatment with resveratrol, 5-FU and/or with TNF-β or with TNF-α resulted in significant synergistic enhancement in inducing caspase-3 cleavage in HCT116 and HCT116R (Figure 5A,B) cells compared to control tumor cultures, suggesting that resveratrol increased TNF-β/5-FU-induced caspase-dependent apoptosis in CRC cells, rather than modulating TNF-β and/or 5-FU-induced NF-κB-regulated apoptotic genes. Taken together, these finding highlights the role of resveratrol in modulating TNF-β and/or 5-FU-induced NF-κB-regulated gene products.

Resveratrol inhibits TNF-β-induced epithelial-to-mesenchymal transition of CRC cells and chemosensitizes to 5-FU in alginate microenvironment cultures

In order to get more insights into the functional suppressive roles of resveratrol in TNF-β-induced tumor malignity in CRC cells and in the process of the synergistic effect with 5-FU in relation to epithelial-to-mesenchymal transition (EMT), we have examined the expression of EMT-associated signaling molecules, such as E-cadherin, vimentin and transcription factor slug (Fig. 5C-D). It has been reported that EMT is related to CSC-like cell formation, up-regulation and development of resistance to chemotherapeutic agents [54] and that it is an essential mechanism for inducing metastasis of cancer cells [50]. It has been described that the transcription factor slug up-regulates the expression of vimentin and down-regulates the expression of E-cadherin [55]. The alginate tumor microenvironment cultures of HCT116 and HCT116R were either left untreated or treated as described in Material and Methods. Untreated control CRC cells (HCT116 and HCT116R) showed a basal expression of E-cadherin, vimentin and slug, and in contrast, the expression of vimentin and slug was markedly up-regulated and E-cadherin was down-regulated in the presence of TNF-β, like TNF-α treatment individually, or in combination treatment (TNF-β or TNF-α with 5-FU) (Figure 5C,D) in alginate cultures. Treatment of CRC tumor microenvironment cultures with 5-FU alone did not change the expression of epithelial/mesenchymal marker E-cadherin, vimentin and the transcription factor slug (Figure 5C,D), highlighting that 5-FU has no effect on the EMT mechanism in CRC cells. However, immunoblotting analysis of CRC tumor microenvironment cultures (HCT116 and HCT116R) treated with resveratrol alone or in combination with 5-FU or with TNF-β or with TNF-α showed marked suppression of vimentin, the transcription factor slug and induction of E-cadherin expression (Figure 5C,D). Taken together, these data clearly provide new insights into how, at least in part, resveratrol can suppress TNF-β-induced tumor metastasis and increasing resistance to chemotherapeutic agents (5-FU) of CRC cells in the 3D-alginate cultures.

## 4. Discussion

The current study was designed to examine, on one hand, the potential pro-inflammatory role of TNF-β (lymphotoxin α) to promote tumour inflammatory microenvironment and thus enhance the malignant potential of CRC cells individually or during treatment with the chemotherapeutic drug 5-FU. On the other hand, it was designed to investigate the potential of resveratrol [56] to block pro-inflammatory pathways activated by TNF-β on the malignant potential of human CRC cells in 3D-alginate tumor microenvironment. Moreover, the pro-inflammatory cytokines have been linked with chronic inflammation and a large body of studies has suggested a reliable coherence between chronic inflammation, which manages the tumor microenvironment and tumorigenesis [9,57,58]. However, the mechanisms of the complex roles of TNF-β and resveratrol molecular signaling during tumorigenesis remain poorly understood.

In this report, we have found the following novel findings: (1) Resveratrol suppressed the TNF-β-, similar to TNF-α-induced invasion and viability in with 5-FU-treated CRC cells in 3D-alginate tumor microenvironment; (2) Resveratrol suppressed TNF-β-, similar to TNF-α-enhanced survival in with 5-FU-treated CRC cells by promoting apoptosis (apoptotic bodies, cleaved caspase-3); (3) Moreover, resveratrol inhibited TNF-β-induced formation of CSC-like cells and chemosensitized CSC to 5-FU; (4) Finally, we showed for the first time, that resveratrol abrogated TNF-β-, similar to TNF-α-mediated expression of NF-κB activation, NF-κB-dependent tumorigenic gene products (MMP-9, CXCR4) and EMT-associated signaling molecules (vimentin, slug, E-cadherin) of HCT116 and HCT116R in alginate tumor microenvironment cultures; highlighting resveratrol as a potent inhibitor of TNF-β-induced tumor malignancy and increasing thereby the chemosensitivity of the CRCs to 5-FU mediated, at least in part through the suppression of NF-κB transcription factor.

We found that TNF-β, similar to TNF-α significantly induced migration and invasiveness of both CRC cell lines from the 3D-based alginate beads matrix. Interestingly, the migration of HCT116R cells was clearly increased compared to the HCT116 adhered cells on the bottom of petri dishes, indicating an increase in cancer malignancy, highlighting, inflammatory cytokines in tumor microenvironment support cancer cell viability and metastasis. Moreover, we have shown that resveratrol alone or in combination with 5-FU suppressed or potentiated the effects of 5-FU through its effects as an anti-metastasis drug on HCT116 and HCT116R cells metastasis in alginate-based 3D culture model. It was noted that 5-FU resistant cells were more sensitive to chemotherapeutic agents, such as resveratrol or the resveratrol and 5-FU combination, represented a potential treatment strategy for 5-FU resistant colon cancer. Indeed, multiple pieces of evidence indicate that development and progression of carcinomas is the result of complex interaction of tumor cells with their immediate microenvironment [4,5]. Activation and misfired inflammation in the tumor microenvironment can promote tumor formation [7]. Indeed, it is recognized that the interaction between the malignant tumor cells and their surrounding microenvironment act as significant mediators triggering tumor growth, invasion and metastasis [59,60,61,62]. Furthermore, several pro-inflammatory cytokines, including members of the Tumor Necrosis Factor (TNF)-superfamily, that are produced in the tumor microenvironment are known to modulate the survival and migration of both tumor and surrounding cells and thus promote cancer development and metastasis [11,12]. Interestingly, TNF-α itself has been shown to regulate the communication between tumor cells, their surrounding stromal cells and the extracellular matrix in several cancers [11]. TNF-α has been suggested to act as an autocrine growth factor and can induce expression of other growth factors. Further, tumor cells often express TNF-α and its receptor, underlining the capacity of autocrine and paracrine stimulation [11,12], thus it has been reported that inflammation is a critical component and is linked to several steps involved in carcinogenesis including tumor survival, metastasis [58,63] and increasing the risk of cancer malignancy [64,65].

Next, we investigated the expression of surface and intercellular molecules linked to cancer stem cell (CSC) markers in human colorectal cancer cells, including CD133, CD44 and ALDH1, which are most widely used to characterize CSC [66,67]. We found with immunofluorescence and immunoblotting examination that expression of the above mentioned CSC markers markedly increased in the presence of TNF-β, similar to TNF-α in CRC cells. This confirmed the above results, that the pro-inflammatory cytokine TNF-β induces the malignant potential of tumor cells by induction of CRC cell activation. Moreover, we could further show that co-treatment with resveratrol significantly suppressed and even diminished stem cell marker expression in both CRC cell lines in alginate microenvironment cultures. It has been reported that CSCs are a subset of a tumor cell population that show stem cell characteristics, including survival in circulation, pluripotency and they are able to move, migrate and form distant metastasis [29,68,69,70]. Furthermore, numerus studies have suggested that CSCs are mainly responsible for initiating cancer cell malignity, resistance and chemotherapy treatment failure and tumor recurrence in a number of tumors including CRC [71,72,73], highlighting the need to develop new strategies for chemosensitivity and overcoming drug resistance targeting CSCs by a combination treatment with chemotherapeutics and resveratrol. It is known that cancer cells can acquire drug resistance by several mechanisms including mutation or overexpression of the drug target, inactivation of the drug or elimination of the drug from the cell [74]. Additionally, CSCs are hypothesized to be naturally resistant to chemotherapy resulting in survival of a small fraction of tumor cells and recurrence [74]. Interestingly, Yu et al. could show that combination of FOLFOX with the natural polyphenol curcumin resulted in marked reduction of colon cancer CSCs [32]. Similarly, Toden et al. could show that epigallocatechin-3-gallate (EGCG), an active catechin present in green tea, specifically targets CSCs in colorectal cancer [31]. Indeed, in our own laboratory we previously showed that the natural polyphenol curcumin modulated the synergistic cross talk in the tumor microenvironment thereby chemosensitizing CRCs to 5-FU and suppressing EMT and CSCs formation [35].

We have shown that resveratrol in combination with 5-FU had a synergistic effect, in increasing the anti-tumor effects of 5-FU including inhibition of invasion and increasing apoptosis in parental HCT116 and HCT116R cells, highlighting that resveratrol chemosensitizes HCT116R cells to 5-FU-based drug regimens. These findings are also in agreement with previous findings that resveratrol exhibits synergistic activity with 5-FU against tumor cells [75]. Diverse drugs may have various signaling targets in tumor cells; thus, if multiple drugs operate on different targets in cells, their combination could result in more potency for suppressing cancer cell growth and the major advantage of resveratrol is that it is non-toxic to healthy cells.

Specific tumor cell death related to the synergistic effects of CRC treatment with a combination of resveratrol and 5-FU was evaluated by multiple parameters, such as DAPI nuclear staining, ultrastructural electron microcopy and apoptosis-associated protein biomarkers. We found that resveratrol significantly induced apoptotic nuclear chromatin condensation and apoptotic bodies in TNF-β-, similar to TNF-α-treated cells. We also further demonstrated that a significant increase in apoptosis and nuclear chromatin condensation with the combination of resveratrol with 5-FU and/or TNF-β or TNF-α compared with either treatment alone in both CRC cell lines. Thus, these results revealed that the resveratrol and 5-FU combination represents a potential treatment option for TNF-β-mediated inflammatory microenvironment on survival and malignity of 5-FU resistant colon cancer.

Several lines of evidence have shown that inflammation stimulates chemokines in the tumor microenvironment, which plays an essential role and is a requisite of both cancer cell promotion and cancer progression [58,63,76]. A large body of evidence has reported that pro-inflammatory cytokines operate mainly due to activation of transcription factor NF-κB, which is able to induce the expression of many genes that have been suggested to down-regulate apoptosis and activate cellular transformation, promotion, invasion, metastasis, survival, chemoresistance, radioresistance and inflammation of the early and late stages of aggressive tumors [8,53,77,78,79]. To study more insight mechanisms of anti-tumor effects of resveratrol signaling on TNF-β-induced malignity, we evaluated whether the NF-κB transcription factor pathway was involved. We have found that NF-κB and certain gene end-products, which are regulated by NF-κB associated with induction of invasion (MMPs), metastasis (CXCR4) and activating those involved in apoptosis (cleavage of caspase-3) are activated in TNF-β-mediated inflammatory microenvironment and tumorigenesis were also modulated by resveratrol. Down-regulation of NF-κB-regulated gene products by resveratrol is at least in part, an important anti-tumor signaling pathway in TNF-β-induced inflammatory microenvironment. Furthermore, it has been reported that Sirt1 protein linked directly with the NF-κB and thus deacetylates the p65 subunit on lysine 310, an essential part for NF-κB transcriptional activity [80,81]. We found that resveratrol enhanced chromatin-condensation and apoptosis was linked with decreasing in p65 protein expression. Consistent with these results, resveratrol suppressed at the same time TNF-β-induced NF-κB-regulated gene end-products. Indeed, our laboratory has shown previously that the ability of the natural compound resveratrol to stimulate Sirt1 activity, to inhibit acetylation of p65 [44] and suppress NF-κB transcription correlates with a sensitization of CRC cells to 5-FU and to apoptosis (activation of capase-3) in TNF-β-mediated tumor inflammatory microenvironment, highlighting resveratrol/Sirt1 pathway serves as a tumor suppressor in CRC cells and can modulate the tumor inflammatory microenvironment induced by other agents. These results are also consistent to those suggested previously in which one of the most important resveratrol signaling pathways is a Sirt1-dependent mode in different kinds of cells and resveratrol is the natural activator of Sirt1 through structural conformational change, resulting in an increased enzymatic activity [82,83,84].

We found major gene products associated with activation of EMT and tumorigenesis were up-regulated (vimentin and slug) and down-regulated (E-cadherin) through TNF-β and resveratrol decreased significantly TNF-β-induced expression of vimentin and this was accompanied with increasing of E-cadherin expression via inhibition of NF-κB and slug activity. These results correlated with the activation of viability and migration and CSC formation induced by TNF-β and 5-FU in tumor microenvironment in both CRC cells. Furthermore, we have found that TNF-β additionally caused dramatically morphological changes on the ultrastructural level, which clearly pointed out a mesenchymal phenotype (Buhrmann et al., unpublished data). Moreover, these findings revealed that deficit of E-cadherin and increase of vimentin causes disruption of cell-cell contact and migration of cancer cells from epithelial assembly to surrounding tissue. Resveratrol promotes cell-cell junction, apoptosis and thus blocks EMT, which is consistent with the results from our laboratory that resveratrol induced an epithelial conversion in CRC cells in tumor microenvironment with decreasing of vimentin and slug and increasing of E-cadherin [44]. Interestingly, these findings are further consistent to those revealed previously that it exists a proportional relationship between tumor malignity (motility, invasion, proliferation, drug resistance) and EMT (increasing mesenchymal signs, vimentin, slug and decreasing of epithelial signs, E-cadherin) [85,86,87,88]. More interestingly, it has been reported that a transient link is established between EMT and the CSC phenotype in cancer cells by cytokines [89].

## 5. Conclusions

**Figure 6:**
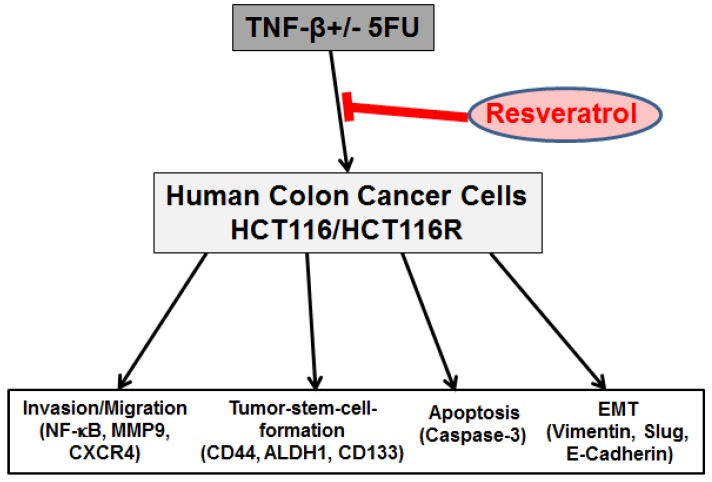
Schematic diagram showing modulatory effect of resveratrol in TNF-β-mediated pro-inflammatory tumor microenvironment on malignity of 5-FU resistant and non-resistant CRC cells.

Overall, our results suggest that resveratrol may suppress multiple pathways activated by TNF-β, modulate the NF-κB-regulated gene expression, CSC formation and increase apoptosis in CRC cells and chemosensitizes CRC cells to 5-FU in TNF-β-induced inflammatory tumor microenvironment (Figure 6). This points out further to the meaningful benefits through combining resveratrol and chemotherapeutic agents for the treatment of CRC.
